# Eczéma de contact allergique au gel d’échographie: à propos d'un cas

**DOI:** 10.11604/pamj.2014.19.129.3660

**Published:** 2014-10-06

**Authors:** Nadia Fihmi, Abdelhafid Elmrahi, Siham Dikhaye, Nada Zizi

**Affiliations:** 1 Service de Dermatologie, CHU Mohamed VI, Faculté de Médecine et de Pharmacie, Oujda, Maroc

**Keywords:** Eczéma de contact allergique, gel d′échographie, tests épicutanés, contact dermatitis, ultrasound gel, tests épicutanés

## Abstract

Le gel d’échographie est un gel utilisé en échographie pour la transmission ultrasonique médicale, il est connu par la plupart des cliniciens pour ces avantages d’être un gel non gras, non irritant, hypoallergénique, incolore et inodore. Cependant, ce gel peut être responsable de la survenue d'un eczéma de contact allergique après son application. Nous rapportons le cas d'une jeune fille qui a présenté une dermite de contact allergique quarante huit heures après la réalisation d'une échographie cervicale.

## Introduction

Les eczémas de contact, encore appelés dermatites de contact, sont des maladies inflammatoires cutanées fréquentes qui surviennent au site de contact avec des molécules chimiques non protéiques. D'exceptionnelles manifestations cutanées allergiques peuvent être observées après application d'un gel d’échographie, nous rapportons une nouvelle observation.

## Patient et observation

**Figure 1:**
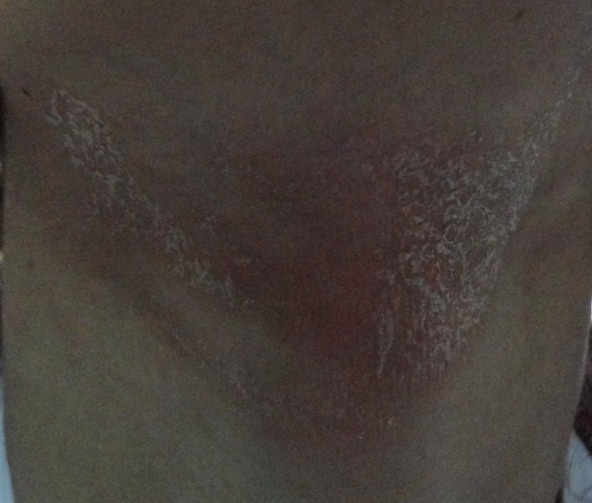
Palacard érythémato-squameux à bords émiettés siégeant au niveau de la partie supérieure de la région cervicale antérieure

**Figure 2:**
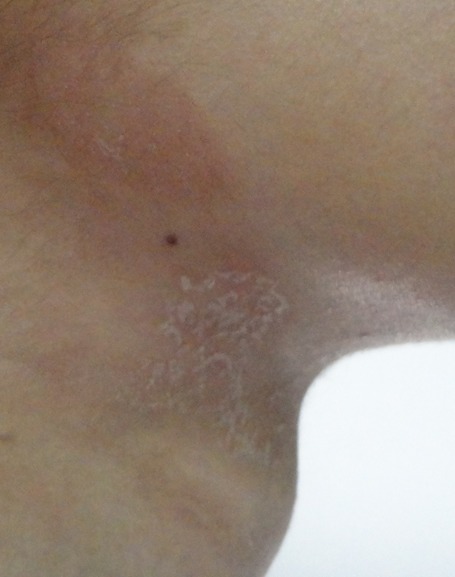
Plaques érythémato-squameuses sur la zone de contact avec le gel d’échographie cervicale

Une jeune fille de 17 ans sans antécédents pathologique notables, notamment pas de dermatite atopique, et il n'y a pas d'antécédents familiaux d'atopie. Elle avait consulté pour une éruption érythémateuse prurigineuse siégeant au niveau du cou survenant 48 heures après application d'un gel de contact pour échographie cervicale. L'interrogatoire ne mettait pas en évidence de prise médicamenteuse ni d'exposition solaire ni application d'un cosmétique ou d'un parfum. À l'examen clinique on notait un placard érythémato-squameux à bords émiettés siégeant au niveau de la partie supérieure de la région cervicale antérieure (Figure 1, Figure 2). L’état général était conservé, il n'y avait pas de fièvre ni adénopathie papable. L'aspect clinique, les délais d'apparition et la localisation ont fait évoquer pour étiologie une allergie au gel d’échographie (Supragel*). La patiente a refusé l'exploration allergologique par patch-tests et aucun test allergologique n'a été effectué. Elle a été traitée par un dermocorticoïde de classe III à raison d'une application par jour pendant une semaine, puis un jour sur deux pendant la seconde semaine. Les signes cutanés locaux ont régressé en quelques jours avec bonne évolution clinique.

## Discussion

L'eczéma allergique de contact (EAC) est une cause majeure des eczémas, il s'agit d'une dermatose inflammatoire dont la prévalence est en constante augmentation. Elle correspond à une réaction immunitaire d'hypersensibilité retardée (HSR) de type IV d'après la classification de Gell et Coombs induite suite au contact répété des individus avec des produits chimiques non protéiques, appelées haptènes, qui sont présents dans notre environnement quotidien: domestique, professionnel et médical [1].

La dermatite de contact allergique (DCA) est causée par une gamme importante de substances chimiques (allergènes potentiels) après un contact prolongé ou répété avec la peau. La fréquence des accidents allergiques par application de gel pour examen échographique qui est de nos jours la technique d'imagerie la plus utilisée [2] serait très faible, quelques cas ont été cependant rapportés dans la littérature (Tableau 1) [3–6].

**Tableau 1:** Cas rapportés dans la littérature d'eczéma de contact au gel d’échographie

Référence :effectif	Aspect lésions	Topographie	Délais apparition	Enquête allergique (gel ; allergène)
Gebhart [3]	Urticaire	Abdomen	48 heures	Gel- Euxyl K400 ++ à 48h
Devemy[3]	Urticaire	Abdomen	Quelques heures	Ultragel* ++ à 48h ; épidermotest –
	Urticaire	Abdomen	24 heures	Sofragel*+ à 48h ; épidermotest –
Tomb[3]	Eczéma	Thorax	24 heures	Aquasonic* +++ à 48h, épidermotest –
	Eczéma	Jambe	48 heures	Spfragel* +++ à 48h ;propylène glycol ++
	Eczéma	Abdomen	36 heures	Spfragel* +++ à 48h ;propylène glycol +
	Rash	Cou	48 heures	Aquasonic* +++ à 48h ; épidermotests-
Ayadi[3]	Eczéma	Cou	24 heures	Aquasonic* +++ ; propylène glycol ++ à 96h
Illy[3]	Eczéma	Cou, thorax	24 heures	Aquasonic* ++ à 48h; épidermotest-
Bourlet[3]	Eczéma	Abdomen	24 heures	Augotgel* à 48h et 96h épidermotest -
Ripert [4]	Eczéma	Mains et poignets	Rythmé par activité professionnelle	Gel-Euxyl K100 ++
Horiguchi[5]	Eczéma	Abdominal	48 heures	Ultra/Phonic Conductivity Gel* ++ + à 48h propylene glycol ++
Khan[6]	urticaire	Périorbitaires	2 heures	Henleys ultrasound gel, épidermotest -

Ces lésions correspondent à une dermite de contact par hypersensibilité à l'un des composants du gel à savoir les conservateurs dont le plus souvent en cause serait le propylène glycol qui n'est pas retrouvé dans la composition de tous les gels, les gélifiants, les stabilisateurs et les colorants [3]. Schématiquement, cet eczéma survient dans quarante huit à soixante douze heures environ après le contact comme l'illustre notre cas, sauf s'il s'agit d'une primo-sensibilisation dans ce cas, le temps d'apparition de l'eczéma avoisine les dix jours [7]. La localisation cervicale s'oriente plus vers une allergie aux cosmétiques, aux vernis à ongles, aux produits volatils (parfums, peintures, végétaux); ce qui n'est pas le cas de notre patiente qui a développé la réaction cutanée suite à l'application d'un gel pour échographie cervicale, ceci suppose que l'interrogatoire est un élément très important de l'enquête étiologique.

L'isolement de l'allergène n'est pas toujours possible du faite de la composition variée du gel et surtout la concentration de ces divers constituants. Le Supragel est constitué d'eau purifiée, glycol-polyacrylate dont Le pouvoir sensibilisant est très controversé et le potentiel irritant varie suivant les acrylates [8], La batterie standard ne contenant pas d'acrylates,ne sont donc testés que les patients chez lesquels l'anamnèse fait suspecter une exposition professionnelle ou domestique aux acrylates. La responsabilité de glycol-acrylate, n'a pas pu être mise en évidence dans notre observation du faite du refus de la patiente de faire les patch test cutanés par manque de moyen. Le traitement symptomatique repose sur l'application de corticoïdes locaux et l’éviction de l'allergène qui est une condition indispensable à la guérison.

## Conclusion

L'allergie de contact au gel d’échographie qui est largement utilisé reste rare mais doit être connue en raison de l'importance des réactions cliniques qui peuvent être très pénible pour les patients et les médecins devraient être conscients de cette possibilité car, il peut affecter davantage la gestion médicale. La prévention des récidives consiste à utiliser un autre gel lors des échographies ultérieures après avoir isolé l'allergène responsable.
